# Immunohistochemical expression of epidermal growth factor receptor (EGFR) in oral squamous cell carcinoma in relation to proliferation, apoptosis, angiogenesis and lymphangiogenesis

**DOI:** 10.1186/1758-3284-2-13

**Published:** 2010-06-25

**Authors:** Seta A Sarkis, Bashar H Abdullah, Ban A Abdul Majeed, Nazar G Talabani

**Affiliations:** 1 Department of Oral Pathology, College of Dentistry, University of Baghdad, Iraq; 2 Department of Pathology, College of Medicine, University of Baghdad, Iraq; 3 Department of Oral Pathology and Medicine, College of Dentistry, University of Sulaimani, Iraq

## Abstract

**Objectives:**

Squamous cell carcinoma (SCC) is by far the most common malignant neoplasm of the oral cavity. A number of etiologic factors have been implicated in its development. During the past few decades, a particular focus has been placed on the investigation of valid biomarkers predictive of cancer behavior and cervical lymph node metastasis in head and neck Squamous cell carcinoma (HNSCC).The present study was designed to investigate the expression of epidermal growth factor in these tumors in relation to proliferation, apoptosis, angiogenesis and lymphangiogenesis.

**Materials and methods:**

Immunohistochemical (IHC) evaluation of epidermal growth factor receptor (EGFR) expression in 40 retrospective OSCC specimens and its correlation with proliferating cell nuclear antigen (PCNA), antiapoptotic antibody (P53), vascular endothelial growth factor (VEGF), and D2-40 monoclonal antibodies (Mab), in relation to the clinicopathological parameters.

**Results:**

Data revealed positive EGFR immunoreactivity in 35(87.5%) cases. There was a statistically significant correlation regarding EGFR extent score with respect to intratumoral lymphatic vessel density (ILVD) (r = 0.35) as well as EGFR intensity score with respect to ILVD and peritumoral lymphatic vessel density (PLVD) (r = 0.33, r = 0.36 respectively). EGFR expression was not correlated with the clinicopathological parameters. Conclusions: EGFR is expressed by most of the cases. EGFR correlation with D2- 40 positive lymphatic vessels suggests a higher tendency of OSCC for lymphatic dissemination. Lack of correlation among the studied markers suggests their independent effect on tumor behavior.

## Background

Oral squamous carcinogenesis is a multistep process in which multiple genetic events occur that alter the normal function of oncogenes and tumor suppressor genes (tsg). Cancer related genes have to be considered in the context of six fundamental changes [1].

•	Self sufficiency in growth signals

•	Insensitivity to growth inhibitory signals

•	Evasion of apoptosis

•	Limitless replicative potential

•	Sustained angiogenesis

•	Ability to invade and metastasize

All normal cells require stimulation on the basis of signals to undergo growth, differentiation and proliferation; many of which carried by growth factors [1,2]. EGFR plays an important role in the differentiation and morphogenesis of many organs and proliferation and survival in mammalian cells [3,4]. EGFR has been reported to be expressed in a variety of human tumors of epithelial origin; over expression of EGFR has been documented in 80% of SCC [1].

Angiogenesis is a crucial step in the successful growth, invasion and metastasis of tumors, without which tumors will not grow more than 1-2 mm^3 ^in diameter [5,6]. VEGF has been considered as a leading candidate in the process of tumor angiogenesis. Various studies reported upregulation of VEGF in different malignancies [7,8].

Tissue growth depends on both cell proliferation and the rate of cell death. PCNA is a 36 kd intra nuclear polypeptide protein whose expression is associated with DNA synthesis and cell proliferation. Many studies demonstrated an association of high expression rate of PCNA with poor prognosis in solid tumors [2,9,10].

Apoptosis is a process of programmed cell death, it is as essential as cell growth for the maintenance of homeostasis [2,11]. P53 is a well known protein that regulates cell cycle check points and is responsible for maintaining the integrity of genome. Mutation of p53 tsg is one of the best known and by far the most frequent genetic alteration identified in malignant tumors [12].

Metastasis unequivocally signifies that a tumor is malignant. Lymphangiogenesis which refers to the growth of new lymphatic vessels has long been regarded as a putative efficient pathway to neoplastic metastasization [13,14]. A new selective immunohistochemical marker is D2-40 which is specific for lymphatic endothelium since it doesn't stain vascular endothelium.

Tumors vary considerably in their behavior, notably in the rate of their growth, the degree of their differentiation and the ability to invade and metastasize. Because of the obscure and variable behavior of cancer, this study concerned different aspects of tumor dynamics through the immunohistochemcial evaluation of EGFR expression in OSCC and its correlation with proliferation, apoptosis, angiogenesis and lymphangiogensis via evaluating PCNA, p53, VEGF and D2- 40 Mabs immunohistochemically.

## Methods

The study sample consisted of 40 retrospective OSCC specimens from the department of oral pathology, college of Dentistry, Baghdad University. An immunoshitochemcial staining with five types of Mabs was preformed: anti EGFR & VEGF (Dako Cytomation -Denmark), anti PCNA, anti P53 and anti D2-40 lymphatic endothelial marker (Dako Cytomation - USA). Negative and positive control slides were included in each IHC run (as recommended by the manufacturers).

### Immunohistochemistry staining procedure

All tests were carried out on 5 μm formalin fixed paraffin embedded sections. Slides were baked in hot air oven at 65°C overnight. Sections were sequentially dewaxed through a series of xylene, graded alcohol and water immersion steps. Antigen (Ag) retrieving was done as recommended by the manufacturers using 500 mL of citrate buffer solution pH.6.0 for p53, pH 8.0 for VEGF and D2-40, on a hot plate at temperature of (95-99°C), while this step was omitted for PCNA Ag. Whereas EGFR Ag and target retrieval was performed by pretreating tissue sections using proteinase K proteolytic enzyme for 10 minutes. Then endogenous peroxidase activity was blocked followed by blocking the non- specific staining. Primary Abs (100 ml) was applied for each section. A dilution of 1:25 for both EGFR and VEGF was used; 1:100 for D2-40, while PCNA and p53 Mabs were ready to use.The samples were then incubated at 4°C overnight in a humid chamber. After washing with PBS, secondary Abs were applied to the sections, incubated and rinsed with a stream of PBS. Primary Abs was visualized with DAB chromogen. Sections were counterstained with Mayer's hematoxyline for 30 seconds, dehydrated and mounted.

### Assessment of IHC results

In each tissue section five representative fields were selected for EGFR, VEGF, PCNA and p53 Mabs and D2-40 positive tumor cells with an average of 1000 tumor cell per case and 200 tumor cells per field.

The immunoreactivity in tumor cells were classified and scored as follows: -(0-25%), + (26-50%), ++ (51-75%), +++(76-100%) for both PCNA and p53 [15]. ± (0-19%), + (20-39%), ++ (40-59%), +++ (60-100%) for VEGF [16]. The extent of EGFR immunostaining was graded and scored as follows: 0 points for negative staining of the considered cells, (1) <10%, (2)10-50%, (3)51-80% and (4) ≥ 80% positive staining of the considered cells. The intensity of staining was scored as 0, no staining; 1, weak; 2, moderate; 3, strong [4,17] -< (10%), +(10-25%), ++(26-50%), +++(51-100%) for D2-40 positive tumor cells [18].

D2-40 positivity was evaluated by selecting six "hot spots" intratumorally and peritumorally. Lymphatic vessel density (LVD) was expressed as the number of stained vessels per optical field [18]. The average number of positively stained vessels in each region was evaluated and recorded separately as intratumoral and peritumoral lymphatic vessel density (ILVD and PLVD). The average count of positive vessels of both regions was recorded together representing the total LVD (TLVD) for each case. In addition, intratumoral (It) and peritumoral (Pt) lymphatic vessel invasion (LVI) was considered evident if at least one tumor cell cluster was clearly visible inside a D2-40 positive vessel [18,19].

Mann-whitney test was used to explore the statistical difference in median between two study groups. The statistical significance, direction and strength of linear correlation were measured by spearman's rank linear correlation coefficient. P value less than the 0.05 level of significance was considered statistically significant.

## Results

### Clinicopathological data

The study sample consisted of 27 males (67.5%) and 13 females (32.5%) with an age range (24-86) years. Clinically, ulcer represented the most frequent clinical presentation (50%) of the cases. The majority of the cases (45%) were seen in the tongue. T_1 _and T_2 _were presented in 12(31.6%) cases for each. Only 12 cases (30.8%) were node positive. Most of the cases (43.2%) were TNM stage III, and were histologically moderately differentiated carcinoma (50%).

### Evaluation of IHC results

**Figure 1:**
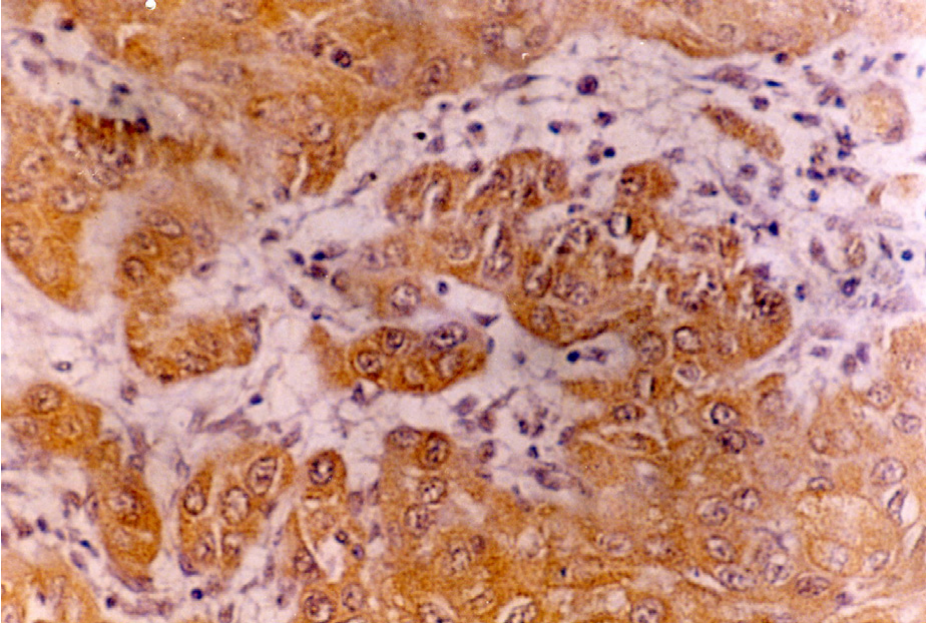
Positive brown membranous and or cytoplasmic EGFR immunostaining in well differentiated SCC (buccal mucosa, tongue and floor of mouth) (x100)

**Figure 2:**
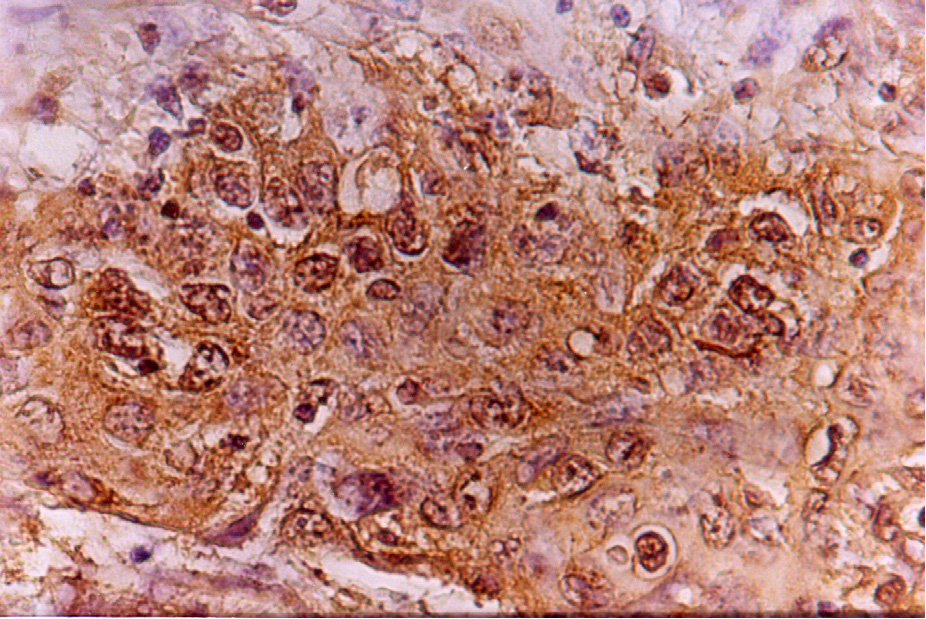
Strong granular cytoplasmic VEGF immunostaining in moderately differentiated SCC (x200)

**Figure 3:**
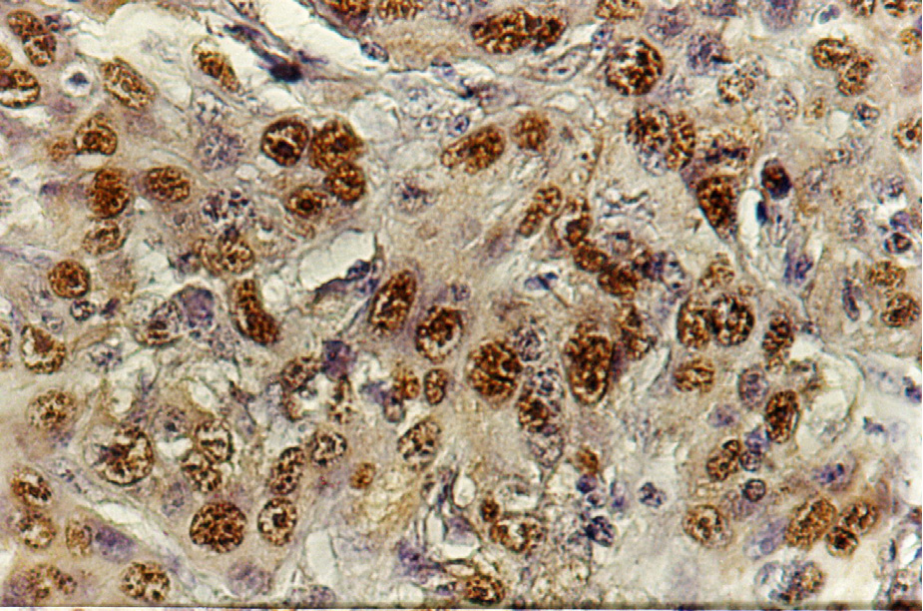
Strong PCNA nuclear immunostaining in moderately differentiated SCC (tongue and floor of mouth (x200)

**Figure 4:**
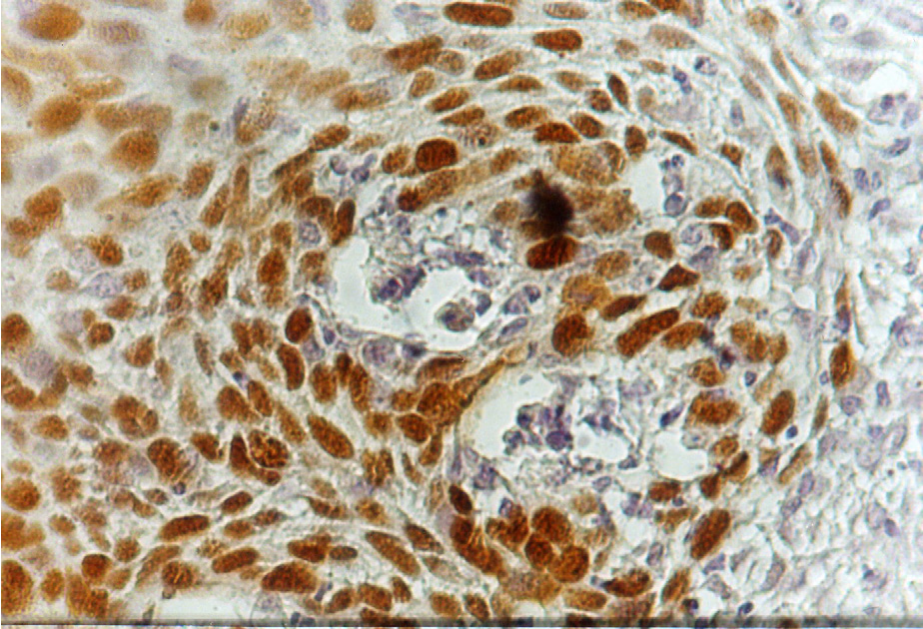
Strong P53 nuclear immunostaining localized to the cells surrounding tumor nests (moderately differentiated SCC-tongue) (x200)

The positivity rate of the selected immunostains EGFR (Fig.1), VEGF (Fig.2), PCNA (Fig.3), P53 (Fig.4); D2-40 in the total sample is shown in (table 1) which reveals positive immunostaining in most of the cases.

**Table 1:** The positivity rate of selected immunostains in the total sample

Positive immunostain (n = 40)	No	%
EGFR immunostain	35	87.5

VEGF immunostain	35	87.5

PCNA immunostain	34	85

P53 immunostain	21	52.5

D2-40 immunostain for tumor cells	15	37.5

Peritumoral LV stain	34	85

Intratumoral LV stain	28	70

Total LV stain	35	87.5

LV= Lymphatic vessel

### Assessment of EGFR expression

Thirty five cases (87.5%) showed positive brown membranous and/or cytoplasmic EGFR immunostaining. In OSCC specimens the expression involved all the epithelial layers while in normal oral mucosa it was localized to the basal cell layer. EGFR positive cells were often seen localized at the periphery of tumor nests. Besides EGFR extent score, the intensity score was also considered. Relative frequency distribution revealed that (37.5%) of the cases showed low intensity (35%) moderate and only (15%) showed high intensity.

### Assessment of D2-40 immunostainnig

**Figure 5:**
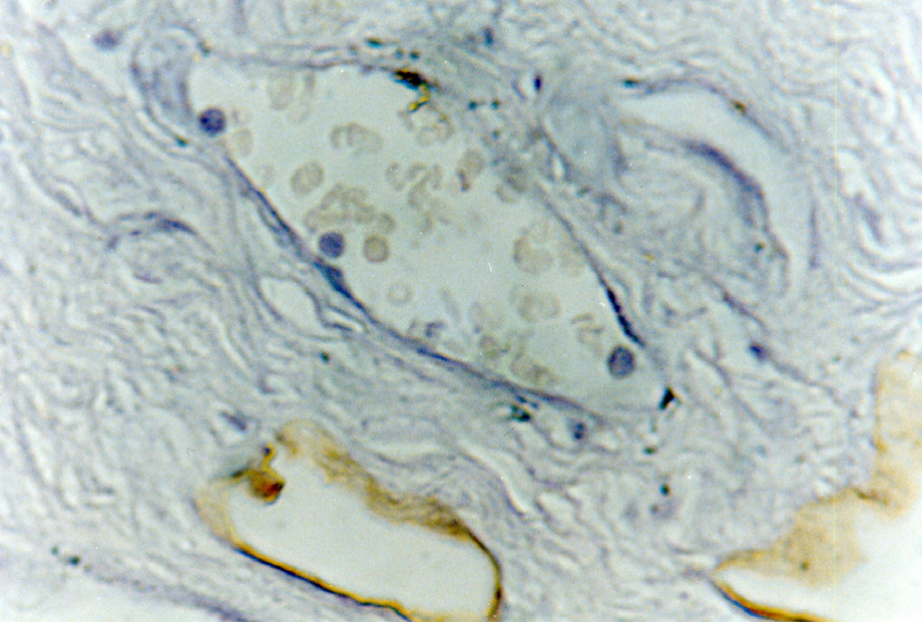
**D2-40 positive lymphatic vessels.** The adjacent blood vessel is negative (arrow) (moderately differentiated SCC-tongue) (x200)

D2-40 brown staining of lymphatic endothelial cells was observed intratumorally, peritumorally or both. Positive lymphatic vessels were unevenly distributed throughout the tumor and their number in the (Pt) area was slightly higher 34(85%) than that in the (It) area 28(70%).The total lymphatic vessel stain was seen in 35 (87.5%) cases. The D2-40 stained lymphatic vessels, the adjacent blood vessels were always unstained (Fig.5).

**Figure 6:**
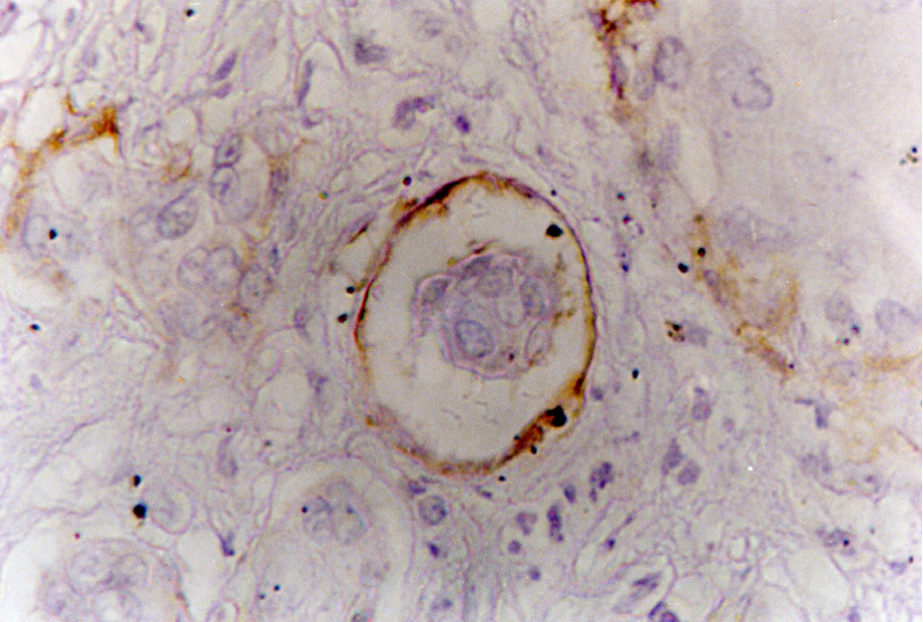
D2-40 positive intratumoral lymphatic vessel containing tumor emboli (poorly differentiated SCC-buccal mucosa) (×200)

Moreover, cancer cells were occasionally observed in (It) lymphatic vessels and/or (Pt) lymphatic vessels (Fig.6), LVI was detected in 13 (37.1%) cases out of 35 D2-40 positive cases of which only five cases presented nodal metastasis, eleven cases were grade II, one of the remaining two was grade I and the other was grade III.

**Figure 7:**
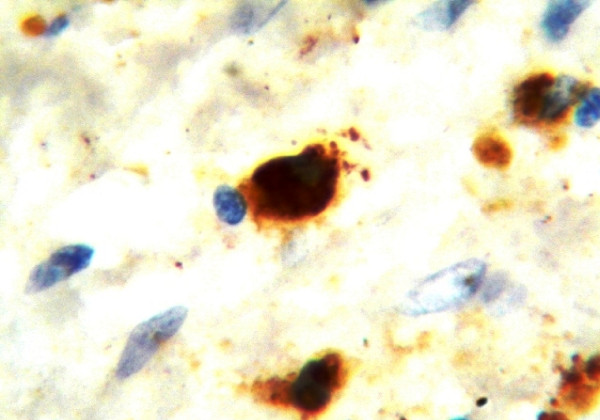
Strong D2-40 tumor cells immunostaining within the stromal tissue indicating single cell microinvasion (arrows) (moderately differentiated SCC- mandible) (x1000)

There was diffuse or strong granular cytoplasmic and/or membranous D2-40 expression in the malignant epithelial cells of 15 (37.5%) cases out of 35 D2-40 positive cases the remaining 28 cases were totally devoid of staining. Eleven of the positive cases were grade II and the remaining four were grade I. In addition, 7 of D2-40 positive tumor cell cases presented LVI. Furthermore, careful examination of D2-40 expressing sections under oil immersion revealed microinvasion of positive malignant cells into the stromal tissue (Fig.7)

### Assessment of the biological markers in relation to the clinicopathological parameters as well as to each other

Mann-whitny's test showed a statistically non significant difference regarding tumor stage, size, lymph node metastasis and grade with the median expression of all the study markers. The only exception was a highly significant PLVD as well as a statistically significant TLVD differences were found with respect to tumor grade (p = 0.002, p = 0.028) respectively (table 2). Moreover a highly significant ILVD and TLVD and a statistically significant PLVD difference was found with respect to LVI (p = 0.002, p = 0.004, p = 0.026) respectively (table 3).

**Table 2:** The difference in median score of selected immunostains by tumor grading (Mann-Whitney)

	Tumor grading		
	**Grade I**	**Grade II-III**	**P**

**Intratumoral LVD**			**0.24**^***[NS]***^

Range	(0 - 200)	(0 - 160)	

Median	18	30	

Interquartile range	(0 - 38)	(8 - 57)	

N	17	23	

Mean rank	18	22.35	

**Peritumoral LVD**			**0.004**

Range	(0 - 155)	(0 - 240)	

Median	15	60	

Interquartile range	(0 - 48)	(35 - 100)	

N	17	23	

Mean rank	14.29	25.09	

**Total LVD**			**0.028**

Range	(0 - 250)	(0 - 300)	

Median	46	93	

Interquartile range	(1 - 78)	(48 - 173)	

N	17	23	

Mean rank	15.79	23.98	

LVD = lymphatic vessel density

**Table 3:** The difference in median score of selected immunostains by lymphatic vessel invasion (Mann-Whitney)

	Lymphatic vessel invasion		
	**Negative**	**Positive**	**P**

**Intratumoral LVD**			**0.002**

Range	(0 - 200)	(24 - 160)	

Median	17	45	

Interquartile range	(0 - 31)	(32 - 64)	

N	22	13	

Mean rank	13.82	25.08	

**Peritumoral LVD**			**0.026**

Range	(0 - 155)	(9 - 240)	

Median	42	72	

Interquartile range	(19 - 62)	(48 - 120)	

N	22	13	

Mean rank	15.05	23	

**Total LVD**			**0.004**

Range	(2 - 250)	(54 - 300)	

Median	52	115	

Interquartile range	(36 - 110)	(91 - 200)	

N	22	13	

Mean rank	14.18	24.46	

LVD = Lymphatic vessel density

On the other hand, spearman's rank linear correlation coefficient test revealed a statistically significant correlation in PCNA vs p53 (r = 0.34), EGFR extent score vs ILVD (p = 0.35), EGFR intensity score vs ILVD, PLVD and TLVD (p = 0.33, p = 0.36, p = 0.36) respectively. In addition D2-40 positive tumor cells showed a statistically significant correlation vs ILVD and TLVD (p = 0.39, p = 0.32) respectively, ILVD vs PLVD and TLVD (p = 0.62, p = 0.85) respectively, PLVD vs TLVD and tumor grading (p = 0.92, p = 0.43) respectively (p < 0.05) (Table 4).

**Table 4:** Correlation matrix

	PCNA	P53	EGFR extent score	EGFR Intensity score	VEGF	D2-40 positive tumor cells	Intratumoral LVD	Peritumoral LVD	Total LVD
P53	0.34(*)								

EGFR **extent score**	-0.19	0.16							

EGFR **Intensity score**	0.03	0.01	0.25						

VEGF	0.07	0.11	0.18	0.25					

D2-40 **positive tumor cells**	-0.01	0.28	0.1	0.07	-0.09				

Intratumoral LVD	0.07	0.26	0.35(*)	0.33(*)	0.08	0.39(*)			

Peritumoral LVD	0.01	-0.01	0.02	0.36(*)	0.2	0.22	0.62(*)		

Total LVD	0.07	0.11	0.18	0.36(*)	0.15	0.32(*)	0.85(*)	0.92(*)	

Tumor staging	-0.06	0.13	-0.09	0.09	-0.05	-0.26	-0.09	0.04	-0.04

Tumor grading	-0.08	0.03	-0.01	0.05	0.09	0.15	0.2	0.43(*)	0.32(*)

Tumor size	-0.06	0.01	-0.18	-0.13	-0.03	-0.32(*)	-0.05	0.1	0.04

Lymph node staging	-0.02	0.22	0.05	0.25	0.03	-0.08	-0.01	0.12	0.06

Age	0.31	0.31	0.07	0.03	-0.2	0.08	-0.04	-0.28	-0.15

## Discussion

It has been reported that the majority of head and neck cancer, including oral cancer express EGFR [20]. Since most oral cancers are epithelial in origin, it is reasonable that they should have a high probability of expressing EGFR. In this series most of the examined cases (87.5%) showed positive EGFR immunostaining. This finding is consistent with previous reports regarding the immunostaining and expression of EGFR in OSCC and other cancers [4,17,20].

A well controlled balance of cellular differentiation and proliferation is necessary for the development and maintenance of normal epithelia throughout the body; since OSCCs are epithelial malignancies, therefore, they should have a high probability of expressing EGFR. The present study confirms the observations of others that high EGFR expression is present in OSCCs which suggests that an uncontrolled growth may be mediated by abnormal EGFR expression [21].

EGFR expression extent and intensity scores revealed by most of the study cases suggest that EGFR expressing carcinomas display pathological features of more aggression which may be attributable to the activation of different signaling pathways that control diverse biological processes [4,22].

EGFR expression involved all epithelial layers in OSCC specimens while in normal oral epithelia it was localized to the basal cell layer, similar results were reported by other investigators [20,23]. Since the squamous epithelium keeps a continuous physiological regeneration in normal conditions, so that it is reasonable that the basal cells interpret signals of EGF by binding to EGFR [23], while its expression beyond basal localization in cancerous tissue suggests that a correlation between EGFR and tumor progress may exist.

The expression was mainly localized to the peripheries of tumor nests, this observation is in accordance with other studies [20,23]. This finding confirms the presence of this receptor on undifferentiated cells and explains that the staining reaction varies with cellular differentiation. Moreover, it may explain that peripheral tumor cells receive a signal from EGF resulting in the proliferation of cancerous tissues.

D2-40 positive lymphatic vessels were recorded in 35 cases, similar results were found in OSCC and other cancers [18,19,24,25], both peritumorally and intraumorally which suggests that these vessels could be a conduit for carcinoma cells and may contribute to lymph node metastasis.

LVI was observed in 13 cases in the current study. Other investigators reported similar findings in OSCC and other cancers [19,24,25]. This may reflect a significant role of these vessels in producing a possible route for the spread of tumor cells to regional lymph nodes.

Fifteen cases showed D2-40 expression by tumor cells, unfortunately there is no enough information concerning D2-40 expression in OSCC, therefore it is difficult to explain these results clearly. However, it may indicate a more aggressive disease phenotype and suggest that D2-40 may be implicated in the differentiation of SCC. Furthermore, D2-40 positive tumor cells were detected in the stromal tissue as well which suggests that it could act as a good marker for microinvasion in OSCC, finding worth more verification.

The current study showed no statistically significant differences between EGFR expression median scores and the clinicopatholgical findings; similar observations were reported in other studies [4,17]. Furthermore, it was not correlated with the other markers as well (except D2-40) which indicate the independent effect of this marker on epithelial cancers development and growth. Moreover, the lack of correlation between EGFR extent or intensity scores in respect to VEGF expression but its existence in respect to LVD as shown in this study would favor lymphatic metastasis of OSCCs rather than hematogenous.

The results of the present study showed a statistically significant difference regarding LVI with respect to the median scores of ILVD, PLVD and TLVD. This finding probably reflects the close relationship between LVI and the lymphatic vessels since carcinomatous cells invade the lymphatic vessels which exist in the area to get an access to the regional lymph nodes. Furthermore, lack of correlation between LVD, LVI and D2-40 positive tumor cells may be attributed to the small size of node positive cases (12 out of 40).

Among all the available studies reviewed, to the best of our knowledge, the present work is the first of its kind in studying comprehensively these biomarkers all together, except several studies that assessed only two or three of them together [15,21,26,27].

The results of this study clarify that the behavior of OSCC is not dependent on a single factor but its combination of multiple biological processes which are independent of each other i.e. malignancy follows no rules.

## Competing interests

The authors declare that they have no competing interests.

## Authors' contributions

SAS: Implementation of the immunohistochemical procedures, immunohistochemical interpretation and statistical analysis. BHA: Study design, histological examination and grading and immunohistochemical interpretive calibration. BAAM: Technical assistance and logistic support during the immunohistochemical procedures. NGT: Histopathological evaluation of the cases, preparation of the article for publication and peer reviewing the final draft. All authors read and approved the final manuscript.
